# Supplementary material for: Phylogenetic Reclassification of Metarhizium granulomatis and Metarhizium viride Species Complex
Source: Pathogens. 2025 Jul 29;14(8):745. doi: 10.3390/pathogens14080745 (PMC12389597; doi:10.3390/pathogens14080745)
Supplement: Supplementary file 1 [file pathogens-14-00745-s001.zip › pathogens-3723802-supplementary.pdf]

Supplementary Materials

Table S1. Measurement of DNA quality of 23 frozen samples of *Metarhizium* (*M.*) *granulomatis* and *M. viride* by nanodrop spectrophotometry (NanoPhotometer® NP80, Implen GmbH, Munich, Germany).

Isolates	DNA concentration [ng/ µl]
VS17118	10.2
VS17809	4.9
VS17814	7.1
VS17813	5.9
VS18415	10.3
VS14512	7.9
VS15807	9.1
VS17810	14
VS18409	2.5
VS17706	6.8
VS18005	4.9
VS18413	17.3
VS18414	7.5
VS17602	3.5
VS10221	11.1
VS8419	3.7
VS16620	14.6
VS9323	8.3
VS16715	5.7
VS18006	9.8
VS2302	2.9
VS17602	4.8
VS17615	6.1

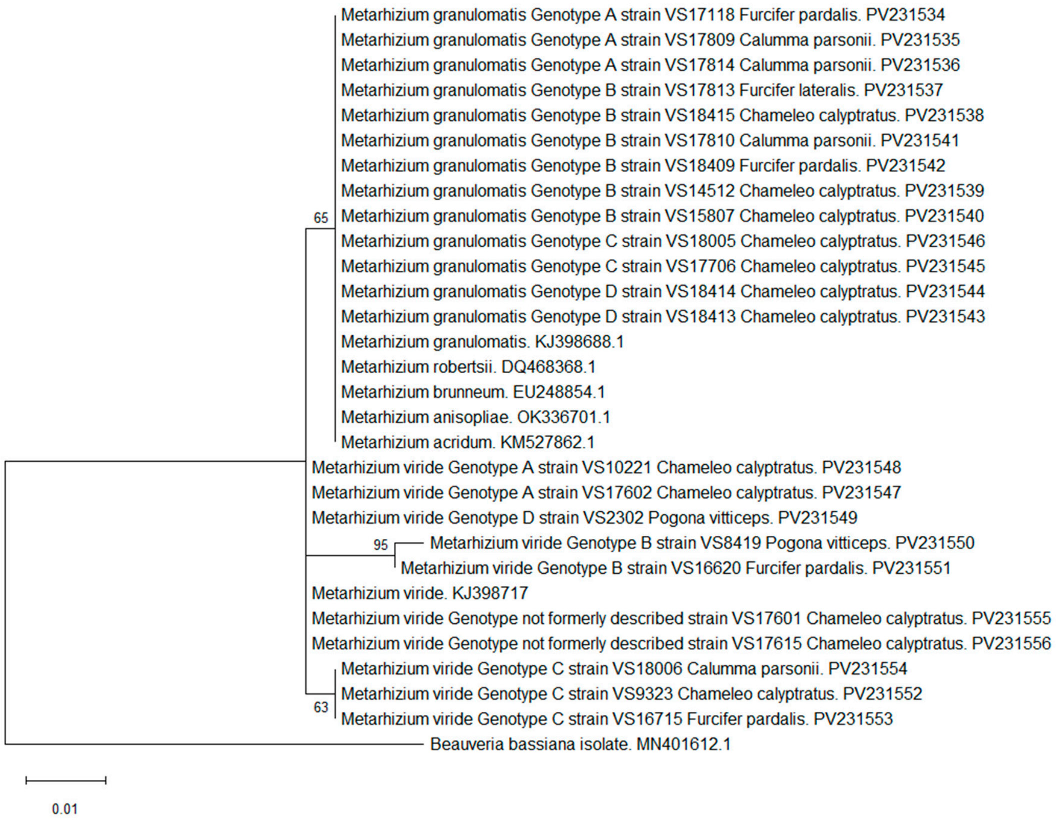

Figure S1. Phylogeny inferred from the amino acid analysis of 13 NRPB2 sequences (RPB1) of *Metarhizium* (*M.*) *granulomatis*—complex and 10 NRPB2 sequences (RPB1) of *M. viride*—complex. Other isolates of *Claviciptitaceae* (Sordariomycetes: Hypocreales) and *Beaveria bassiana* were added as additional outgroup taxa. The phylogenetic model presents 23 newly generated NRPB2 (RPB1) fragments (NCBI Acc.-Nr. PV231534-PV231556). Reference sequences with accession numbers were taken from the GeneBank database (http://www.ncbi.nlm.nih.gov) and NCBI Acc.-Nr. are shown.

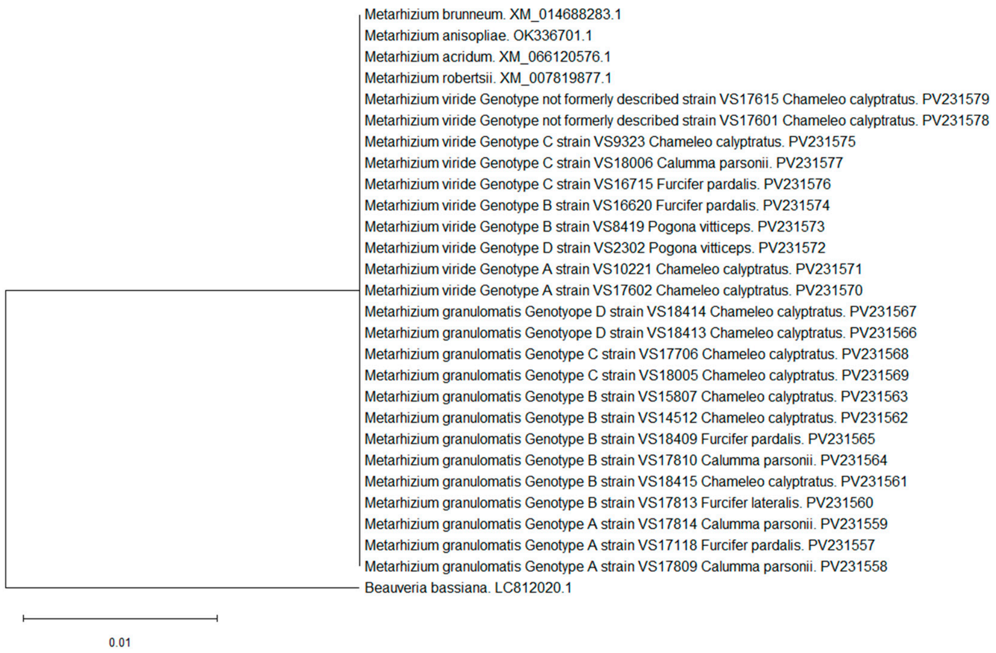

Figure S2. Phylogeny inferred from the amino acid analysis of 13 NRPB2 sequences (RPB2) sequences of *Metarhizium* (*M.*) *granulomatis*—complex and 10 NRPB2 sequences (RPB2) sequences of *M. viride*—complex. Other isolates of *Claviciptitaceae* (Sordariomycetes: Hypocreales) and *Beaveria bassiana* were added as additional outgroup taxa. The phylogenetic model presents 23 newly generated NRPB2 (RPB2) fragments (NCBI Acc.-Nr. PV231557-PV231579). Reference sequences with accession numbers were taken from the GeneBank database (http://www.ncbi.nlm.nih.gov) and NCBI Acc.-Nr. are shown.

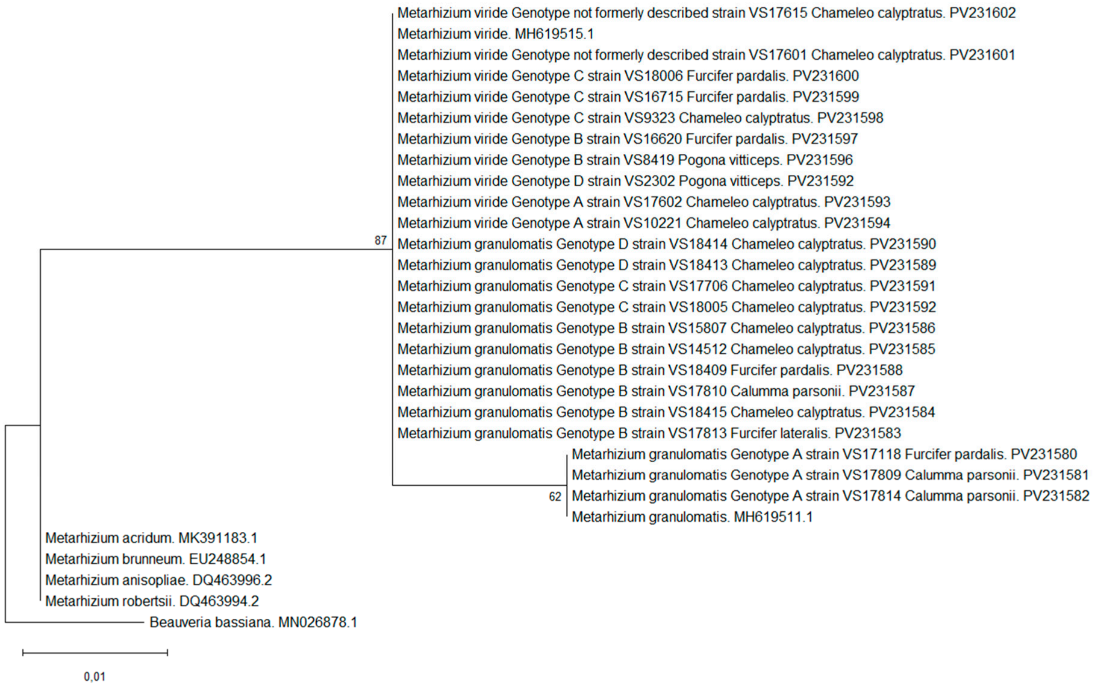

Figure S3. Phylogeny inferred from the amino acid analysis of 13 EF1α sequences of *Metarhizium* (*M.*) *granulomatis*—complex and 10 EF1α sequences of *M. viride*—complex. Other isolates of

Claviciptitaceae (Sordariomycetes: Hypocreales) and *Beaveria bassiana* were added as additional out-group taxa. The phylogenetic model presents 23 newly generated EF1α fragments (NCBI Acc.-Nr. PV231580-PV231602). Reference sequences with accession numbers were taken from the GeneBank database (http://www.ncbi.nlm.nih.gov) and NCBI Acc.-Nr. are shown.

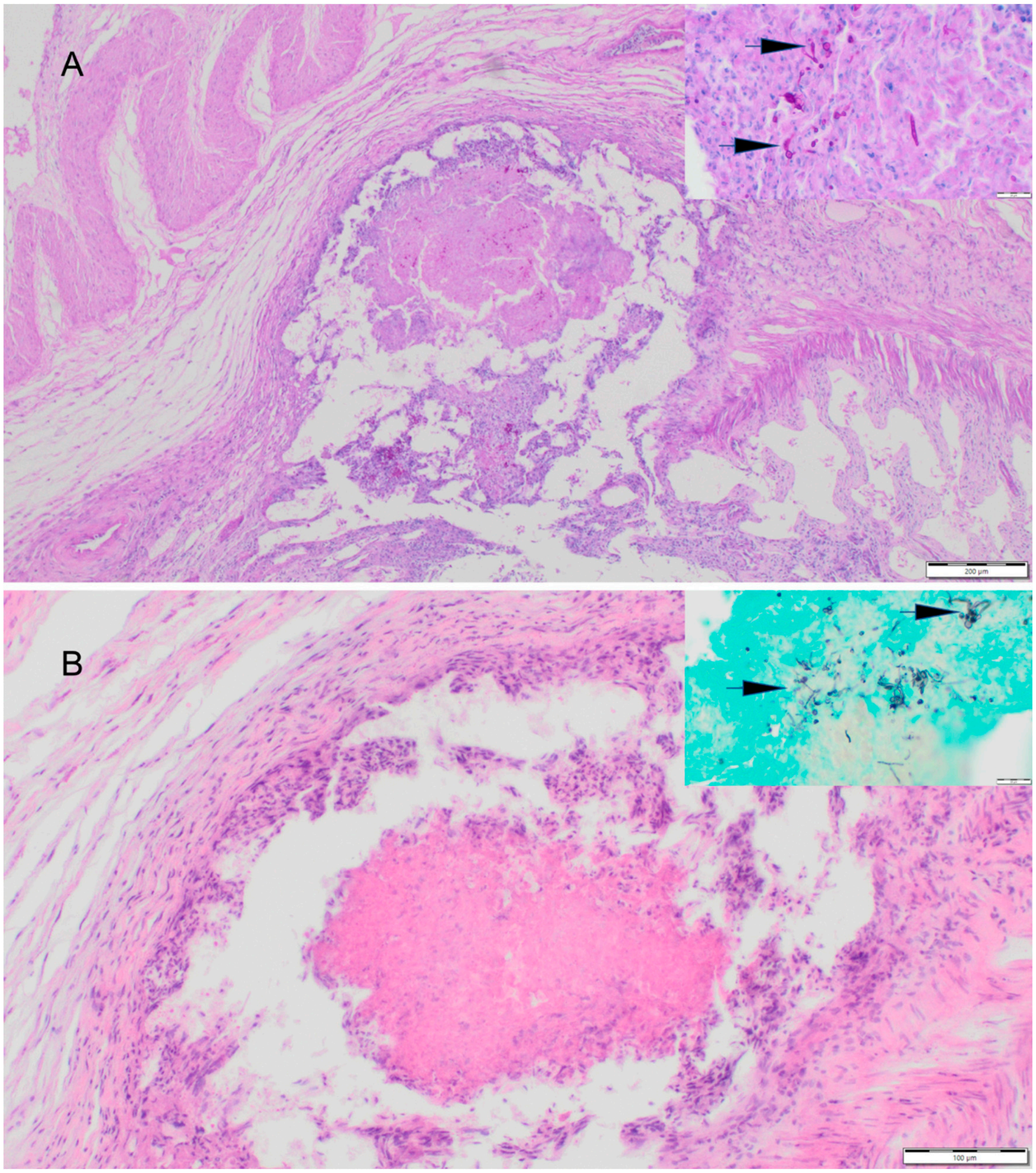

Figure S4. Microphotograph of the cloaca of a veiled chameleon (*Chamaeleo calyptratus*) with a granulomatous mycotic colorectitis. (**A**) PAS, 40x magnification, inlay 400x magnification, black arrows showing the PAS-positive fungal hyphae and conidia inside the fibrinous granuloma. (**B**) H&E, 100× magnification, inlay GMS, 400× magnification, black arrows showing the fungal hyphae and conidia inside the fibrinous granuloma.
